# Polydopamine-Modified Copper Coordination Mesoporous Silica Nanoparticles Loaded with Disulfiram for Synergistic Chemo-Photothermal Therapy

**DOI:** 10.3390/pharmaceutics16040512

**Published:** 2024-04-07

**Authors:** Junhong Ling, Yingying Cai, Haozhan Feng, Zhen Liu, Xiao-kun Ouyang

**Affiliations:** School of Food and Pharmacy, Zhejiang Ocean University, Zhoushan 316022, China; lingjunhong@zjou.edu.cn (J.L.); caiyingying@zjou.edu.cn (Y.C.); fenghaozhan@zjou.edu.cn (H.F.); liuzhen@zjou.edu.cn (Z.L.)

**Keywords:** antitumor, nano-delivery system, disulfiram, copper ions, chemo-photothermal therapy

## Abstract

Disulfiram (DSF) degrades to diethyldithiocarbamate (DTC) in vivo and coordinates with copper ions to form CuET, which has higher antitumor activity. In this study, DSF@CuMSN-PDA nanoparticles were prepared using mesoporous silica with copper ions, DSF as a carrier, and polydopamine (PDA) as a gate system. The nanoparticles selectively released CuET into tumor tissue by taking advantage of the tumor microenvironment, where PDA could be degraded. The release ratio reached 79.17% at pH 5.0, indicating pH-responsive drug release from the nanoparticles. The PDA-gated system provided the nanoparticles with unique photothermal conversion performance and significantly improved antitumor efficiency. In vivo, antitumor experiments showed that the designed DSF@CuMSN-PDA nanoparticles combined with near-infrared light (808 nm, 1 W/cm^2^) irradiation effectively inhibited tumor growth in HCT116 cells by harnessing the combined potential of chemotherapy and photothermal therapy; a synergistic effect was achieved. Taken together, these results suggest that the designed DSF@CuMSN-PDA construct can be employed as a promising candidate for combined chemo-photothermal therapy.

## 1. Introduction

Cancer is the second most prevalent cause of mortality worldwide [1]. The most common cancer treatments are surgery, chemotherapy, and radiation [2,3]. Other treatment options include targeted, immunological, and hormonal therapies, as well as stem cell or bone marrow transplants [4,5,6]. As an emerging targeted therapy, nanocarriers directly deliver and release drugs to cancer and tumor cells, thereby reducing their toxicity and side effects [7,8,9,10,11]. Moreover, compared with a single therapy, photothermal therapy delivered through nanocarriers in combination with chemotherapy significantly enhances the efficacy of cancer treatment [12,13,14,15,16].

Disulfiram (DSF) is an FDA-approved pharmaceutical agent that is used to treat chronic alcohol consumption [17,18]. It has been widely used for over 70 years because of its low side effects and price [19,20]. In recent years, DSF has shown significant promise as an anticancer pharmaceutical agent [21,22]. It shows a unique Cu^2+^-dependent mode [22,23]. Under physiological conditions, DSF is degraded into diethyldithiocarbamate (DTC) [24,25]. As a metal chelator, DTC binds Cu^2+^ to form DTC–copper chelate (CuET) [26,27]. The CuET complex induces mitochondrial division, resulting in cell apoptosis [28,29,30]. A substantial quantity of ROS is produced during the chelation reaction between DSF and Cu^2+^, destroying DNA, proteins, and lipids in tumor cells, leading to apoptosis [31,32,33]. Consequently, the CuET complex had a stronger anticancer effect than that of DSF or Cu^2+^ alone [34,35]. Therefore, the addition of Cu^2+^ is expected to increase the efficacy of DSF-based cancer treatments [36,37]. Mesoporous silica nanoparticles (MSNs) are widely used for pharmaceutical drug delivery [38,39,40], molecular imaging, and molecular adsorption because of their large specific surface area, simple preparation, and surface modification [41,42,43]. Polydopamine (PDA) is a synthetic melanin analog with good biocompatibility and excellent photothermal properties [44,45,46,47,48]. The broadband UV, visible, and near-infrared absorption ranges of PDAs have been harnessed in many light-dependent applications [49,50].

**Scheme 1:**
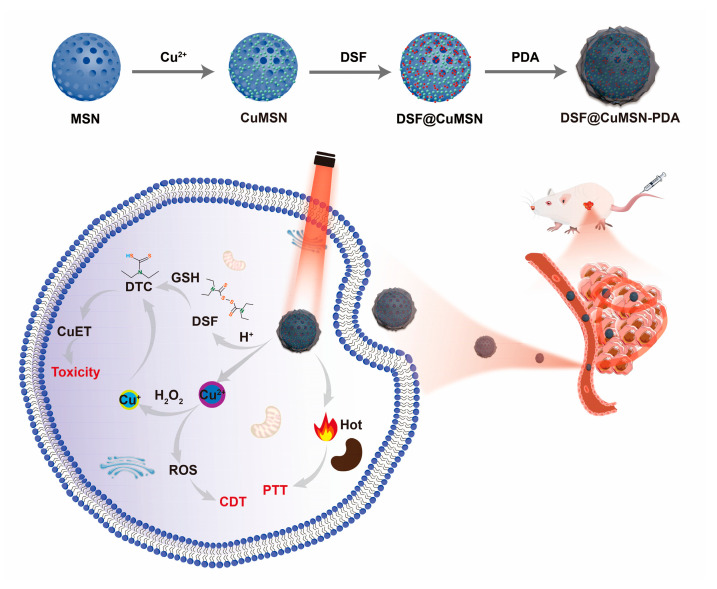
Schematic illustration of the preparation of DSF@CuMSN-PDA and mechanisms related to synergetic antitumor therapy.

In this study, we prepared nanoparticles designated DSF@CuMSN-PDA (Scheme 1) by encapsulating Cu^2+^-chelated MSNs with a pH-responsive PDA loading of the water-insoluble drug DSF. The nanoparticles were characterized via transmission electron microscopy (TEM), scanning electron microscopy, X-ray diffraction (XRD), X-ray photoelectron spectroscopy (XPS), zeta potential analysis, and Fourier-transform infrared spectroscopy (FTIR). The effect of pH on the DSF release rate was also determined. The toxicity of DSF@CuMSN-PDA to normal and tumor cells was investigated using an MTT assay. The uptake of DSF@CuMSN-PDA by the tumor cells was investigated using in vitro cell uptake assays. The anticancer efficacy and selectivity of DSF@CuMSN-PDA were evaluated in vivo in tumor-bearing mice.

## 2. Materials and Methods

### 2.1. Materials

Cetyltrimethylammonium chloride (CTAC), tetraethyl orthosilicate, 2,7-dichlorofluorescein diacetate (DCFH-DA), dopamine hydrochloride, Hoechst 33258, and ethanol butanol were purchased from Aladdin Biochemical Technology Co., Ltd. (Shanghai, China). Dimethyl thiazole blue (MTT) was purchased from Beijing Solarbio Corporation (Beijing, China). DSF, fluorescein isothiocyanate (FITC), and indocyanine green (ICG) were purchased from Macklin Biochemical Co., Ltd. (Shanghai, China). Cupric nitrate trihydrate (Cu(NO_3_)_2_·3H_2_O), ammonia solution (NH_3_·H_2_O), hydrochloric acid (HCl), dimethyl sulfoxide, hydrogen peroxide (H_2_O_2_), triethanolamine, and phosphate buffer saline (PBS) were purchased from Sinopharm Chemical Reagent Co., Ltd. (Shanghai, China). The L929 and HCT116 cells were supplied by the Cell Bank of the Chinese Academy of Sciences (Shanghai, China). Acridine orange/ethidium bromide reagent was purchased from Yuan Ye Biological Co., Ltd. (Shanghai, China). Deionized water was used in this study, and all the compounds utilized in the experiments were of analytical grade, including the aforementioned compounds.

All experiments involving animals were approved by the Animal Ethics Committee of Zhejiang Ocean University (approval No. 2021055). Male BALB/c nude mice (18–22 g) were provided by the Ziyuan Laboratory Animals (Hangzhou, China).

### 2.2. Synthesis of Mesoporous Silica Nanoparticles (MSNs)

MSNs were created using a previously described technique. Two grams of CTAC and 0.071 mL triethanolamine were added to 20 mL deionized water, thoroughly mixed and dissolved, and stirred in an oil bath at 95 °C for 60 min. Thereafter, 1.5 mL of tetraethyl orthosilicate was added for an hour. The reaction mixture was centrifuged at a rotational velocity of 12,000 rpm for 10 min to obtain MSNs. The residue was then cleaned using anhydrous ethanol and H_2_O. Finally, the CTAC template was removed via extraction with HCl–ethanol (1:9, *v*/*v*) at 60 °C for 48 h to further purify the MSN product.

### 2.3. Synthesis of CuMSN

In 40 mL of water, 20 mg of MSNs and 200 mg of Cu(NO_3_)_2_·3H_2_O were mixed and agitated for a duration of 20 min. Subsequently, 6 mL of NH_3_·H_2_O (30 wt%) was included in this mixture. The mixture was transferred to a 100 mL Teflon autoclave. The autoclave was then placed in a heating oven and maintained at 60 °C for 3 h. The resulting products were centrifuged at 12,000 rpm for 5 min.

### 2.4. Preparation of CuMSN-PDA

CuMSNs (10 mg) and dopamine hydrochloride (12.5 mg) were mixed with 10 mL of 10 mM Tris-HCl solution (pH 8.5), dissolved thoroughly, and agitated for 12 h. Subsequently, CuMSN-PDA was formed via centrifugation at 12,000 rpm for 8 min.

### 2.5. Preparation of DSF@CuMSN-PDA

DSF (5 mg) and the CuMSNs (10 mg) were mixed in 10 mL of ethanol. The components were adequately dissolved and agitated at atmospheric temperature for 24 h. Subsequently, the mixture was centrifuged (12,000 rpm, 8 min) to obtain a solid. The solid portion was dissolved in 10 mL of 10 mM Tris-HCl solution (pH 8.5) and then stirred with 12.5 mg dopamine hydrochloride for 12 h. After 12 h, the mixture was centrifuged at 12,000 rpm for 5 min to precipitate the DSF@CuMSN-PDA, which was rinsed with ethanol three times for further application.

### 2.6. Characterization

FTIR spectra were obtained using a Bruker Tensor II spectrometer (Bruker, Karlsruhe, Germany). Scanning electron microscopy (Hitachi S4800, Hitachi, Tokyo, Japan) and TEM (Lorentz JEM-2100, JEOL, Tokyo, Japan) were used for microstructural characterization. Particle size, zeta potential, and PDI were calculated at 25 °C using a Malvern ZS90 laser particle size analyzer (Malvern, Worcestershire, UK). The average diameter, pore size, and specific surface area under nitrogen adsorption–desorption conditions were determined using a Quadrasorb EVO gas adsorption apparatus (Quanta Instruments, Boynton Beach, FL, USA), based on the Brunauer-Emmett-Teller (BET) equation. The elemental content was quantified using energy-dispersive X-ray spectroscopy (EDS) and the phases of the as-synthesized nanoparticles were analyzed using XRD (D8 Advance, Bruker, Karlsruhe, Germany). The elemental compositions and chemical states of the materials were analyzed via XPS (ESCALAB, Thermo Fisher Scientific, Waltham, MA, USA).

### 2.7. In Vitro DSF and Cu^2+^ Release

The release capabilities of DSF from DSF@CuMSN-PDA and Cu^2+^ from the CuMSNs were assessed in vitro. This was performed by placing dialysis bags in PBS buffer at varying pH values (7.4 and 5.0) and subjecting them to agitation at 120 rpm at a temperature of 37 °C. At specific times, 2 mL of the dialysate was sampled and replenished with 2 mL of PBS. The concentrations of DSF and Cu^2+^, which indicated the release capacity, were determined using UV–vis spectroscopy and atomic absorption spectrophotometry, respectively.

### 2.8. Photothermal Properties of DSF@CuMSN-PDA

The photothermal properties of DSF@CuMSN-PDA were evaluated by measuring the temperature changes under near-infrared (NIR; 808 nm) irradiation. The influence of concentration on photothermal performance was also examined by observing changes in the temperature of DSF@CuMSN-PDA at various concentrations (50, 100, and 200 μg/mL). DSF@CuMSN-PDA was subjected to various powers (0.5, 1.0, and 2.0 W/cm^2^) to assess the impact of power on the photothermal characteristics. DSF@CuMSN-PDA (100 μg/mL) was used to record the photothermal stability and heating–cooling was seen under a power intensity of 2.0 W/cm^2^. Solution temperatures were measured using an FLIR C2 infrared thermal imaging camera. The photothermal conversion efficiencies were calculated by referring to a previously reported method [51].

### 2.9. Hemolysis Test

The hemolysis rate is a common method used to evaluate hemocompatibility. The employed method was a slight modification of a previously described method. Briefly, red blood cells were obtained by centrifuging (3500 rpm, 10 min) fresh anticoagulated rabbit blood in PBS. Red blood cells were treated with PBS, deionized water, or various DSF@CuMSN-PDA solution concentrations. After 8 min of spinning at 2000 rpm to collect the remaining liquid, an ultraviolet–visible spectrophotometer was used to measure the optical density at 540 nm. The rate of hemolysis was determined using the following equation: hemolysis rate (%) = [(OD_samples_ − OD_negative control_)/(OD_positve control_ − OD_negative control_)] × 100%.

### 2.10. Intracellular ROS Detection

DCFH-DA was used as a luminous marker to measure the amount of ROS produced by cells. A total of 5 × 10^5^ HCT116 cells were put in 6-well plates. After 24 h, they were treated with different amounts of DSF@CuMSN-PDA (0, 10, 40, and 80 μg/mL) for 4 h. After 20 min of labeling with DCFH-DA, the cells were rinsed thrice with PBS and examined under a fluorescence microscope.

### 2.11. In Vitro Cytotoxicity and Live/Dead Cell Staining Assay

The MTT cell assay was used to assess the cytotoxicity of the nanoparticles. HCT116 and L929 cells were seeded in 96-well plates and grown for one day. DSF, DSF+Cu^2+^ (DC), CuMSN-PDA (CP), DSF@CuMSN-PDA (DCP), and DSF@CuMSN-PDA+Laser (DCPL) were added, and the cells were cultured for one day. In the DCPL group, after growth for 12 h in an NIR laser for 5 min at 808 nm and 1.0 W/cm^2^, the wells were filled with 5 mg/mL of MTT. After 4 h, dimethyl sulfoxide was added as a substitute for the incubation medium. A microplate reader was used to measure the optical density.

An assay to label live or dead cells was performed to evaluate the cytotoxicity of the preparations. After seeding in 6-well plates, L929 and HCT116 cells were treated with PBS, DSF, DC, CP, DCP, or DCPL for 24 h. For the DCPL group, 12 h later, the cells were exposed to an NIR laser with a wavelength of 808 nm and an intensity of 1.0 W/cm^2^ for 5 min. After 12 h, the cells were washed with PBS, and acridine orange/ethidium bromide was added. The cell morphology was observed using a Nikon TI-S fluorescence microscope.

### 2.12. Cellular Uptake

To assess the cellular internalization of the carriers, a modified version, denoted FITC@CuMSN-PDA, was synthesized by substituting DSF with FITC. HCT116 cells were seeded in a six-well plate for 24 h. Subsequently, the cells were exposed to FITC@CuMSN-PDA for 2, 4, 6, and 8 h. The cells underwent three rounds of washing using PBS, were fixed via immersion in a 4% paraformaldehyde solution for 20 min, stained with 1 mL of Honest 33258 (10 μM) for 20 min, and examined using a fluorescence microscope.

### 2.13. Construction of a Tumor-Bearing Mouse Model

A tumor-bearing mouse model was generated using male BALB/c nude mice with an average body weight of approximately 19 g. A subcutaneous implantation procedure was performed on the mice by injecting a 100 µL solution of HCT116 cells (1 × 10^7^) into the right abdomen. The tumor volumes in the mice were measured at regular intervals of two days.

### 2.14. In Vivo Antitumor Effect

As the tumor size approached 100 mm^3^, the mice were allocated to six distinct groups (*n* = 4) using the random allocation method. On days 0, 3, 6, 9, and 12, the mice were injected intravenously with the following: (1) PBS; (2) DSF; (3) DC; (4) CP; (5) DCP; (6) DCPL (50 μL/mouse, 5 mg/kg). The DCPL group was exposed to NIR laser radiation for 5 min at 24 h post-injection. The temperature difference was observed. Data were measured every two days. The tumor volume was calculated using the following formula: tumor volume = (tumor length) × (tumor width)^2^/2. After seven injections, the mice were euthanized, and primary organs were obtained for histological examination using H&E and TUNEL staining.

### 2.15. In Vivo Biodistribution

Nanoparticles were labeled using ICG (ICG@CuMSN-PDA) to visualize their biodistribution. ICG@CuMSN-PDA (50 μL/mouse, 200 μg/mL) was injected via the mouse tail vein, and fluorescence imaging signals were recorded using an AniView600 Imaging System (Biolight Biotechnology, Guangzhou, China) at 0, 1, 4, 8, 12, and 24 h.

### 2.16. In Vivo Biosafety Assay

Biosafety was assessed through biochemical blood analyses. The mice were divided into six groups and then administered PBS, DSF, DC, CP, DCP, or DCPL every two days. Fourteen days later, the mice were sacrificed via cervical dislocation, and blood samples were collected. The samples were centrifuged for 15 min at 1000 rpm, and the plasma was stored at 4 °C. Blood biochemical parameters were measured using a hematology analyzer (XS-500i, SYSMEX, Shanghai, China).

### 2.17. Statistical Analysis

All data are presented as mean ± standard deviation. One-way analysis of variance was performed for multiple group comparisons of means. * *p <* 0.05 was considered statistically significant, and ** *p <* 0.01 and *** *p <* 0.001 were considered highly significant.

## 3. Results and Discussion

### 3.1. Preparation and Characterization of DSF@CuMSN-PDA

**Figure 1:**
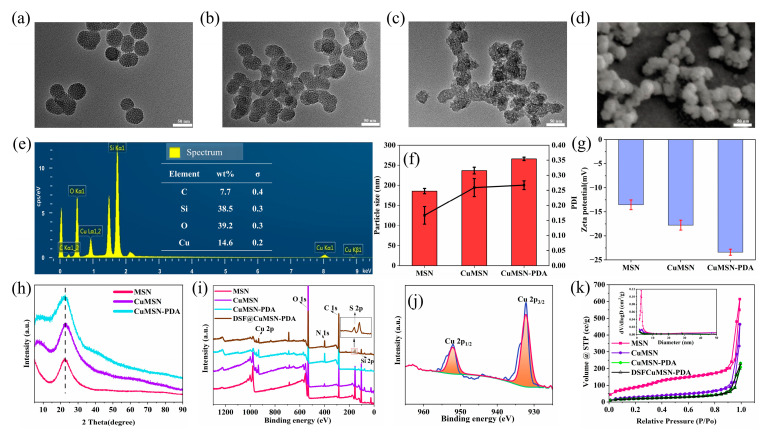
Characterization of MSNs, CuMSNs, and DSF@CuMSN-PDA. (**a**–**c**) TEM images of MSNs, CuMSNs, and CuMSN-PDA. (**d**) SEM image of CuMSNs. (**e**) EDS image of CuMSNs. (**f**) Particle size and PDI of MSNs, CuMSNs, and CuMSN-PDA. (**g**) Zeta potentials of MSNs, CuMSNs, and CuMSN-PDA. (**h**) XRD patterns of MSNs, CuMSNs, and CuMSN-PDA. (**i**) XPS patterns of MSNs, CuMSNs, CuMSN-PDA, and DSF@CuMSN-PDA. (**j**) The XPS raw-data spectrum (Blue) fitted with Cu species (Magenta). (**k**) N_2_ adsorption–desorption isotherms and pore sizes of MSNs, CuMSNs, CuMSN-PDA, and DSF@CuMSN-PDA.

The synthesized MSNs and CuMSNs exhibited a uniform spherical morphology as observed via TEM (Figure 1a,b). The CuMSNs were approximately 50 nm in Figure 1d (Figure S2). The surface of the CuMSN-PDA had a coating layer (Figure 1c), and the particle size increased (Figure 1f), indicating successful PDA coating. The EDS spectrum of the CuMSNs suggests that the relative percentages of O, C, Si, and Cu were 39.2, 7.7, 38.5, and 14.6%, respectively (Figure 1e), indicating the successful synthesis of CuMSNs. Moreover, the zeta potentials of MSNs, CuMSNs, and CuMSN-PDA were determined to be −13.5 ± 1.01, −17.8 ± 1.03, and −23.4 ± 0.64 mV (Figure 1g), attributed to a reduction in the potential by the PDA negative charge when present as surface modification and encapsulation. Moreover, the FTIR spectra confirmed the successful synthesis of MSNs, CuMSNs, and CuMSN-PDA (Figure S1).

XRD patterns (Figure 1h and Figure S3) showed a characteristic mesoporous silica peak (2θ = 22.6) in MSNs, CuMSNs, and CuMSN-PDA, indicating that MSNs were successfully synthesized and that the mesoporous crystallized structure of MSNs was not destroyed after chelation of Cu and encapsulation of PDA, in agreement with the TEM and EDS results. XPS analysis (Figure 1i,j) showed that the binding energy centers of Cu 2p_3/2_ and Cu 2p_1/2_ were at 932.2 and 952.0 eV, respectively, and the binding energy center of S 2p was at 162.6 eV (Figure S4). The results indicated the successful chelation of Cu and loading of DSF.

The N_2_ adsorption–desorption isotherms (Figure 1k) revealed that the CuMSNs exhibited a characteristic Langmuir IV isotherm, as per the IUPAC classification. The surface area of the CuMSNs was determined to be 102.94 m^2^/g, and the measured pore width was 3.05 nm. The pore size distribution of CuMSN changed to 3.0484 nm, indicating that the chelation of Cu^2+^ has a certain influence on the pore size. This surface area enables easy diffusion of small molecules into the interior of the carrier through the pores [52]. In contrast, the surface area of CuMSN-PDA was 70.96 m^2^/g, which was much smaller than that of the CuMSNs, indicating that the application of the PDA coating led to a substantial decrease in the surface area and pore size of the carrier material. This result implies that PDA can function as a blocker, slowly releasing the loaded drugs into biological circulation. Additionally, after DSF loading, the surface area of the complex and the scope of the pores shrank to 1.72 nm and 68.72 m^2^/g, respectively.

### 3.2. In Vitro DSF and Cu^2+^ Release

**Figure 2:**
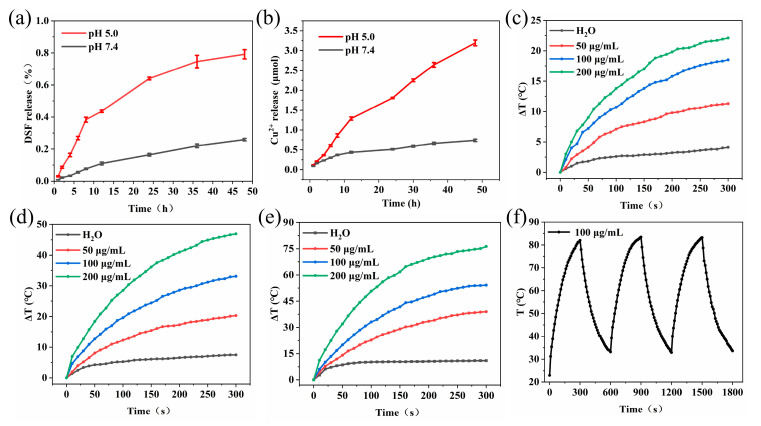
(**a**,**b**) Release profiles of DSF from DSF@CuMSN-PDA and Cu^2+^ from CuMSNs at pH 5.0 and 7.4. (**c**–**e**) Photothermal heating curves of DSF@CuMSN-PDA at different powers (0.5, 1.0, and 2.0 W/cm^2^) and concentrations (50, 100, and 200 μg/mL). (**f**) Photothermal stability of DSF@CuMSN-PDA (100 μg/mL) for three consecutive photothermal cycles under 808 nm laser irradiation (2.0 W/cm^2^).

The DSF release rate from DSF@CuMSN-PDA at pH 5.0 (79.17%, 48 h) was notably higher than that at pH 7.4 (25.89% over 48 h) (Figure 2a). The release of Cu^2+^ in an acidic environment was substantial and sustained, proving the pH-dependent nature of Cu^2+^ release (Figure 2b). These acid-triggered release profiles of DSF and Cu^2+^ stem from the pH-dependent dissolution properties of DSF@CuMSN-PDA. Within this nanoplatform, CuMSNs functioned as nanocarriers, creating a microenvironment rich in Cu^2+^. Additionally, the release results of DSF also include the reasons for the premature leakage of DSF from DSF@CuMSN-PDA before it reaches and accumulates at the designated target sites within the circulatory system [53,54].

### 3.3. Photothermal Properties of DSF@CuMSN-PDA

The rise in the temperature of DSF@CuMSN-PDA upon NIR irradiation at 808 nm was monitored to evaluate its in vitro photothermal conversion capability. During a 5 min irradiation at 0.5 W/cm^2^, the temperature increased with exposure to 50, 100, and 200 μg/mL of DSF@CuMSN-PDA; specifically, the temperature rose from 11.3 to 18.5 and 22.1 °C, respectively (Figure 2c). When DSF@CuMSN-PDA was exposed to NIR irradiation applied at an energy of 2 W/cm^2^ for 5 min, the temperature of DSF@CuMSN-PDA increased rapidly from 0 °C to 76.3 °C. In comparison, the temperature of the control (H_2_O) only increased by 11.0 °C within 5 min. The photothermal effect of DSF@CuMSN-PDA was related to the concentration and irradiation time of the carrier, as indicated by the temperature curves (Figure 2c–e). In addition, when subjected to high-energy laser irradiation (2.0 W/cm^2^), DSF@CuMSN-PDA exhibited a greater increase in temperature than when subjected to low-energy irradiation (Figure 2e). There were no significant changes in carrier performance after three switching cycles, suggesting that the photothermal effect mediated by DSF@CuMSN-PDA could be easily recycled (Figure 2f). The photothermal conversion efficiency (η) of DSF@CuMSN-PDA was calculated to be 23.63% (Figure S5), which exceeds that of previously reported photothermal agents [55]. In summary, DSF@CuMSN-PDA exhibited excellent stability and strong capability for photothermal conversion.

### 3.4. Hemolysis Test

**Figure 3:**
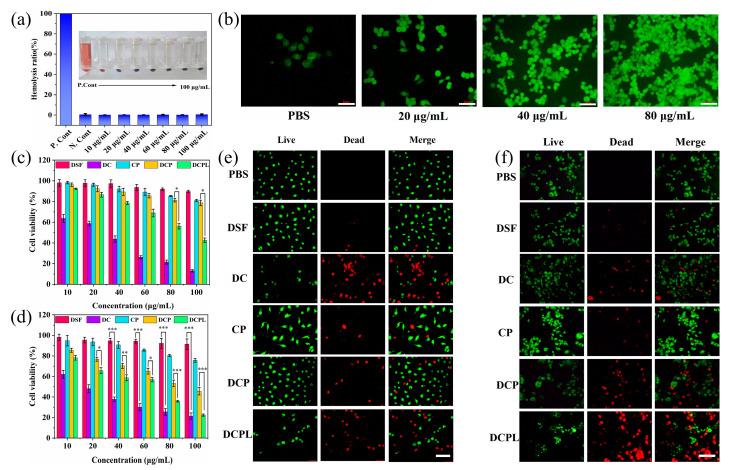
(**a**) Hemolysis rate of DSF@CuMSN-PDA (P. Cont: positive control group treated with saline solution; N. Cont: negative control group treated with deionized water). (**b**) Intracellular ROS generation of HCT116 cells treated with different concentrations of CuMSN-PDA (scale bar: 50 μm). (**c**) Survival rate of L929 cells treated with different formulations for 24 h. (**d**) Survival rate of HCT116 cells treated with different formulations for 24 h (* *p* < 0.05, ** *p* < 0.01, *** *p* < 0.001). (**e**) Live/dead staining of L929 cells treated with different formulations for 24 h (scale bar: 100 μm). (**f**) Live/dead staining of HCT116 cells treated with different formulations for 24 h (scale bar: 100 μm).

At 10–100 μg/mL concentrations of DSF@CuMSN-PDA, the hemolysis rates were approximately 0.2% (Figure 3a), much lower than 5%, showing that the tested DSF@CuMSN-PDA has excellent hemocompatibility and can be used for intravenous injection.

### 3.5. Intracellular ROS Detection

An ROS-sensitive probe, DCFH-DA, was employed for the fluorescence detection and evaluation of the ROS generation capability of CuMSN-PDA. DCFH-DA is prone to hydrolysis by intracellular esterases to produce DCFH and oxidize ROS, and green fluorescence is observed under a microscope. Significantly enhanced cells exhibited a more pronounced emission of green fluorescence when treated with high concentrations of CuMSN-PDA because the Cu^2+^ ions released from CuMSN-PDA in the slightly acidic environment of HCT116 cells react with excess H_2_O_2_ to generate ROS (known as a Fenton-like reaction). These results indicate that CuMSN-PDA induced ROS generation under mildly acidic conditions in a concentration-dependent manner.

### 3.6. In Vitro Cytotoxicity and Live/Dead Cell Staining Assay

The MTT-based cytotoxicity assay data related to the nanoparticles are shown in Figure 3c,d. The viability of the CP- and DCP-treated cells was over 80%, indicating that the nanocarriers were not significantly toxic to normal cells and were, therefore, highly biocompatible. In contrast, the cell viability of 100 µg/mL DC was less than 25%. This is due to the fact that Cu(DTC)_2_ within the DC group possesses high water solubility, enabling it to penetrate cell membranes and exert a cytotoxic effect on cells.

The presence of DSF enabled the production of a more potent CuET chelate than DSF. As the viability of DCPL-treated (100 µg/mL) cells decreased to 42.63%, when coupled with NIR excitation at 808 nm, DCPL nanoparticles with NIR irradiation effectively inhibited the growth of the tested cells. The live/dead cell staining results were similar to those of the MTT assay (Figure 3e,f), further confirming the bioactivity of the tested constructs. Here, aridine orange (AO) and ethidium bromide (EB) fluorescent dyes were used for double staining. AO is capable of penetrating cells that have an intact membrane and intercalating with nuclear DNA, resulting in the emission of bright green fluorescence. In contrast, EB can only penetrate cells with compromised membranes; upon binding to nuclear DNA within these cells, it emits an orange–red fluorescence. Cells undergoing apoptosis typically display rounded or condensed morphologies along with aggregated structures, which are associated with intensified staining and more pronounced fluorescence.

### 3.7. Cellular Uptake

FITC-labeled nanoparticles were used as tracers to evaluate the cellular uptake of DSF@CuMSN-PDA. At 0 h, no green fluorescence was detected in the visual field. By 2 h, partial release of FITC occurred due to decomposition of the outer layer. At 8 h, an abundance of FITC fluorescence was evident within the cells (Figure S6). The intensity of green fluorescence obtained from FITC showed a positive correlation with the incubation time, indicating that FITC@CuMSN-PDA possesses good time-dependent cellular uptake efficiency and tumor-targeting ability.

### 3.8. In Vivo Antitumor Effect

**Figure 4:**
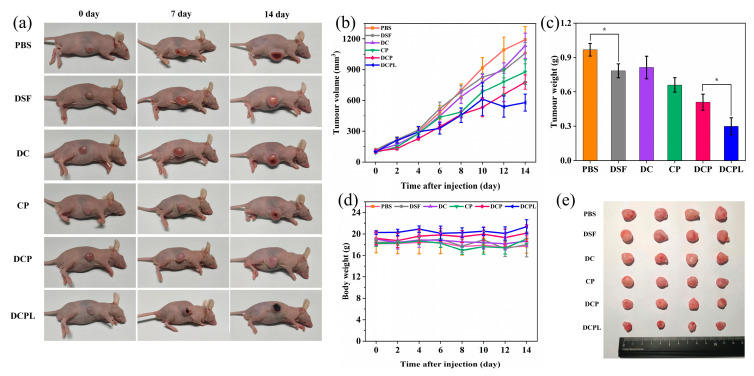
(**a**) Photographs of representative mice from different groups as captured on days 0, 7, and 24. (**b**) Tumor volumes of HCT116 tumor-bearing mice after different treatments. (**c**) Tumor weights of HCT116 tumor-bearing mice after various treatments (* *p* < 0.05). (**d**) Body weights of HCT116-tumor-bearing mice after different treatments. (**e**) Digital photographs of excised tumors following different treatments on day 14.

The tumor volume, tumor weight, and body weight of tumor-bearing mice were recorded for 14 days. (Figure 4a–e). Both DC and DSF exhibited minimal tumor suppression when compared to the PBS group, while CP demonstrated a slight reduction in tumor growth. The therapeutic efficacy of the DCP treatment was not as pronounced as that observed with DCPL, which can be attributed to the influence of light activation. Meanwhile, compared to the DSF, DC, CP, DCP, and DCPL groups, the DCPL group experienced a reduced rate tumor volume increase (*p* < 0.05). In particular, after 7 days, several tumors in the DCPL group ceased growth, presenting the smallest among all groups (*p* < 0.05). The body weights of the groups did not differ significantly from one another. These results suggest that DSF@CuMSN-PDA has an excellent ability to treat cancer and exhibits good biocompatibility.

**Figure 5:**
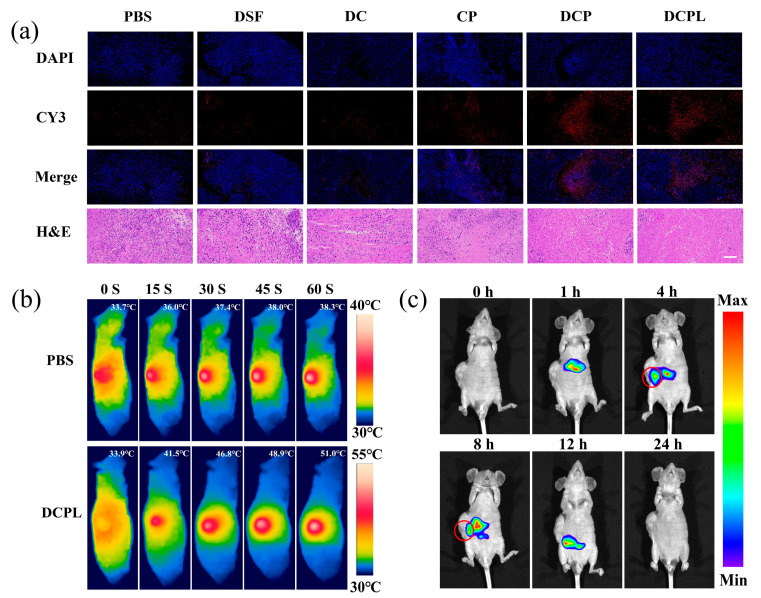
(**a**) TUNEL and H&E staining of tumor tissues after various treatments (scale bar: 100 μm). (**b**) Infrared thermal images of PBS and DCPL under 808 nm laser irradiation (1.0 W/cm^2^, 5 min). (**c**) Fluorescence image of mice with HCT116 tumors after ICG@CuMSN-PDA was injected into their veins.

H&E staining showed no noticeable tissue damage in the major organs across any of the treatment groups compared to the PBS group (Figure S7), indicating low systemic toxicity and good biocompatibility of the drug system. H&E staining also indicated a larger number of large-sized nuclei in tumor tissues treated with PBS than in those treated with DCP or DCPL (Figure 5a). TUNEL staining (Figure 5a) showed significant apoptosis in the DCPL group, supporting evidence for the synergistic effect of combining chemotherapy and photothermal therapy using DSF@CuMSN-PDA. 

When the tumor was exposed to NIR radiation at a wavelength of 808 nm and power of 1.0 W/cm^2^, infrared thermal imaging revealed that the tumor region of the DCPL group had a marked increase in temperature (from 33.9 to 51.0 °C) over the course of the 60 s exposure. However, the PBS group showed only a minor increase in temperature (from 33.7 to 38.3 °C), indicating that significant in vivo light–heat conversion occurs in DSF@CuMSN-PDA upon NIR irradiation (Figure 5b).

### 3.9. In Vivo Biodistribution

When the tumor volume reached approximately 100 mm^3^, ICG@CuMSN-PDA was injected through the tail vein. One hour after injection, the spleen and liver contained the highest accumulation of ICG@CuMSN-PDA. After 4 h, notable accumulation of ICG@CuMSN-PDA was observed at the tumor site, persisting even after 12 h (since fluorescence was still detected near the tumor), indicating a targeted accumulation of ICG@CuMSN-PDA at tumor sites, highlighting its selectivity and biosafety.

### 3.10. In Vivo Biosafety Assay

**Figure 6:**
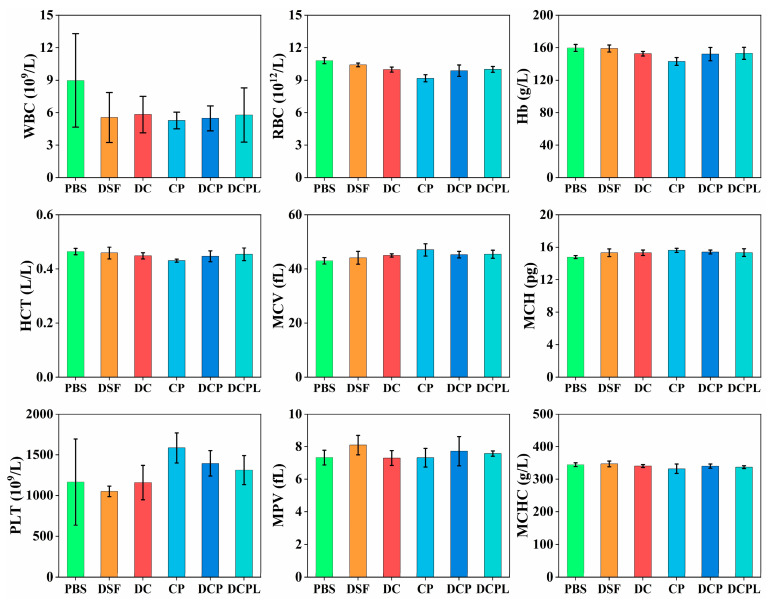
Variations in blood indices on day 14 of HCT116 mice with tumors that received various formulations.

Blood samples from the mice in each group were collected and routinely tested. The measured blood indicators did not significantly differ among groups (Figure 6). These findings support the in vivo biosafety and stability of the designed nanoparticles.

## 4. Conclusions

In summary, a DSF-loaded, PDA-modified nano-delivery system, DSF@CuMSN-PDA, was constructed for synergistic chemo-photothermal anticancer therapy. DSF@CuMSN-PDA selectively delivered DSF to tumors, where Cu and DSF were simultaneously released in an acid-dependent manner. These nanoparticles act as a vector system, creating a microenvironment with high Cu^2+^ expression and enhancing the anticancer activity of DSF. In vivo antitumor experiments demonstrated that the combination of DSF@CuMSN-PDA with infrared light irradiation effectively suppressed the growth of HCT116 mouse tumors while maintaining good biosafety. This study lays the foundation for the development of DSF nanotherapeutics with combined chemo-photothermal properties for cancer treatment.

## Data Availability

The data presented in this study are available in this article (and Supplementary Materials).

## Supplementary Materials

The following supporting information can be downloaded at: https://www.mdpi.com/article/10.3390/pharmaceutics16040512/s1, Figure S1. Infrared spectra of DSF, MSN, CuMSN, CuMSN-PDA, DSF@CuMSN-PDA. Figure S2. SEM image of CuMSN. Figure S3. XRD patterns of MSN, CuMSN, and CuMSN-PDA. Figure S4. Peak fitting pattern of S. Figure S5. The photothermal conversion efficiency (η) of DSF@CuMSN-PDA (100 μg/mL) under 2.0 W/cm^2^ laser irradiation with 808 nm. Figure S6. Fluorescence images of cellular uptake of FITC@CuMSN-PDA after 0 h, 2 h, 4 h, and 8 h incubation (scale bar: 100 μm). Figure S7. H&E staining of major organs after different treatments.
