# Supplementary material for: Polydopamine-Modified Copper Coordination Mesoporous Silica Nanoparticles Loaded with Disulfiram for Synergistic Chemo-Photothermal Therapy
Source: Pharmaceutics. 2024 Apr 7;16(4):512. doi: 10.3390/pharmaceutics16040512 (PMC11054823; doi:10.3390/pharmaceutics16040512)
Supplement: Supplementary file 1 [file pharmaceutics-16-00512-s001.zip › pharmaceutics-2906920-supplementary.pdf]

pharmaceutics

Supplementary Information

Polydopamine Modified Copper Coordination Mesoporous Silica Nanoparticles Loaded with Disulfiram for Synergistic Chemo-Photothermal Therapy

Junhong Ling, Yingying Cai, Haozhan Feng, Zhen Liu, Xiao-kun Ouyang *

School of Food and Pharmacy, Zhejiang Ocean University, Zhoushan 316022, China;
* Correspondence: xkouyang@zjou.edu.cn; Tel.: +86–580–2554781

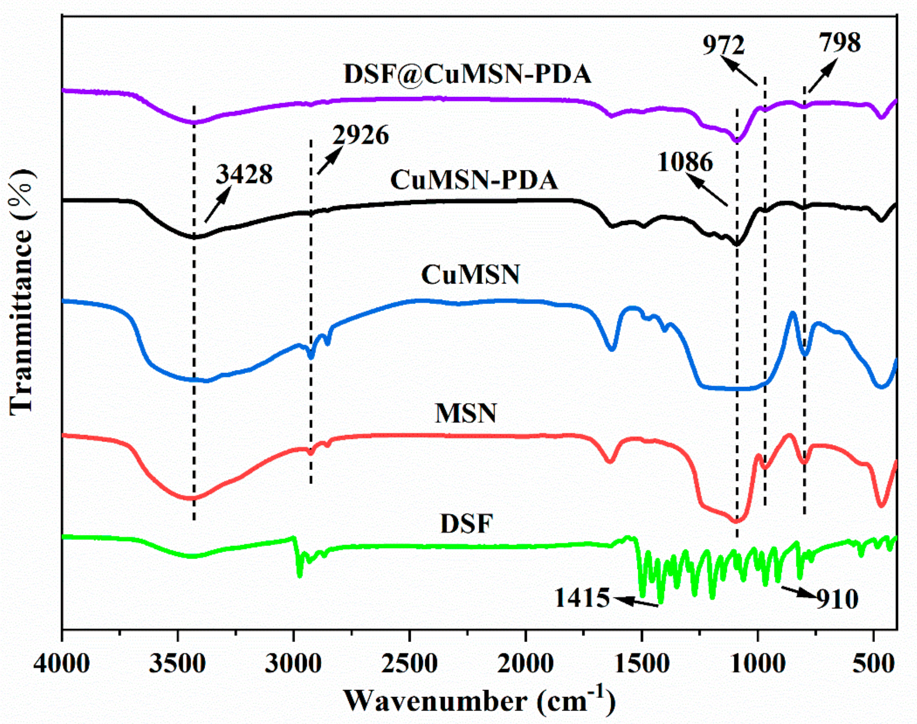

Figure S1. Infrared spectra of DSF, MSN, CuMSN, CuMSN-PDA, DSF@CuMSN-PDA.

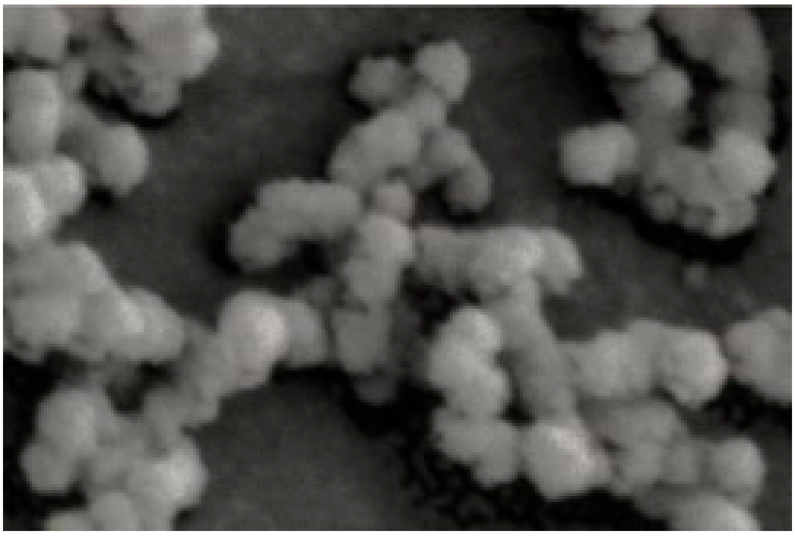

Figure S2. SEM image of CuMSN.

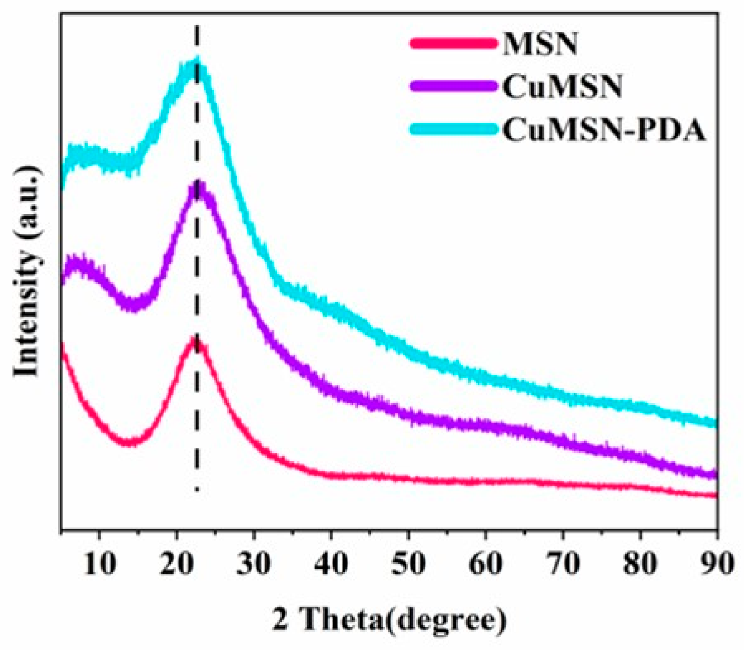

Figure S3. XRD patterns of MSN, CuMSN, and CuMSN-PDA.

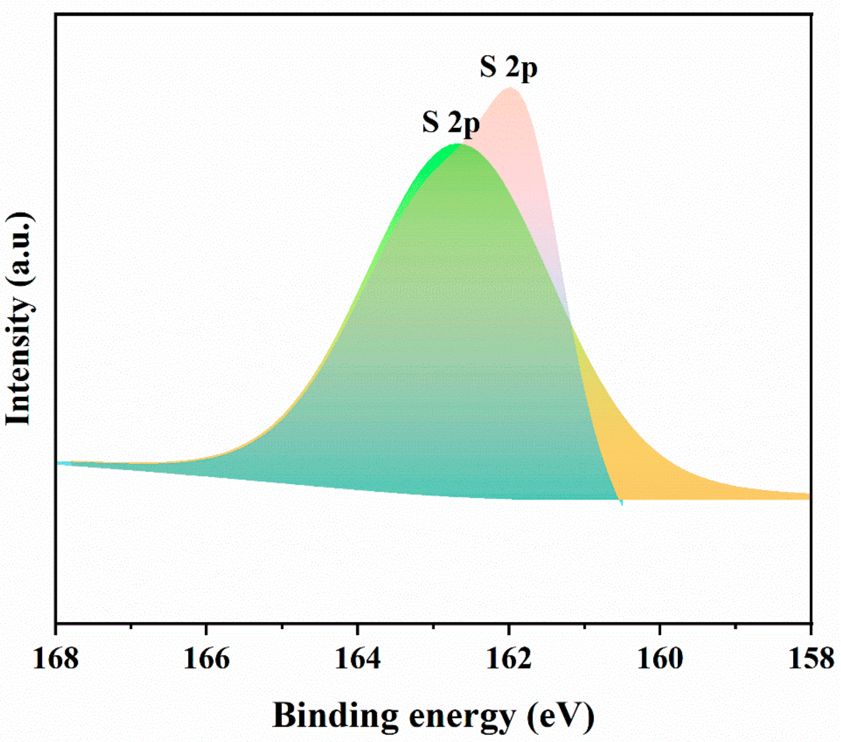

Figure S4. Peak fitting pattern of S.

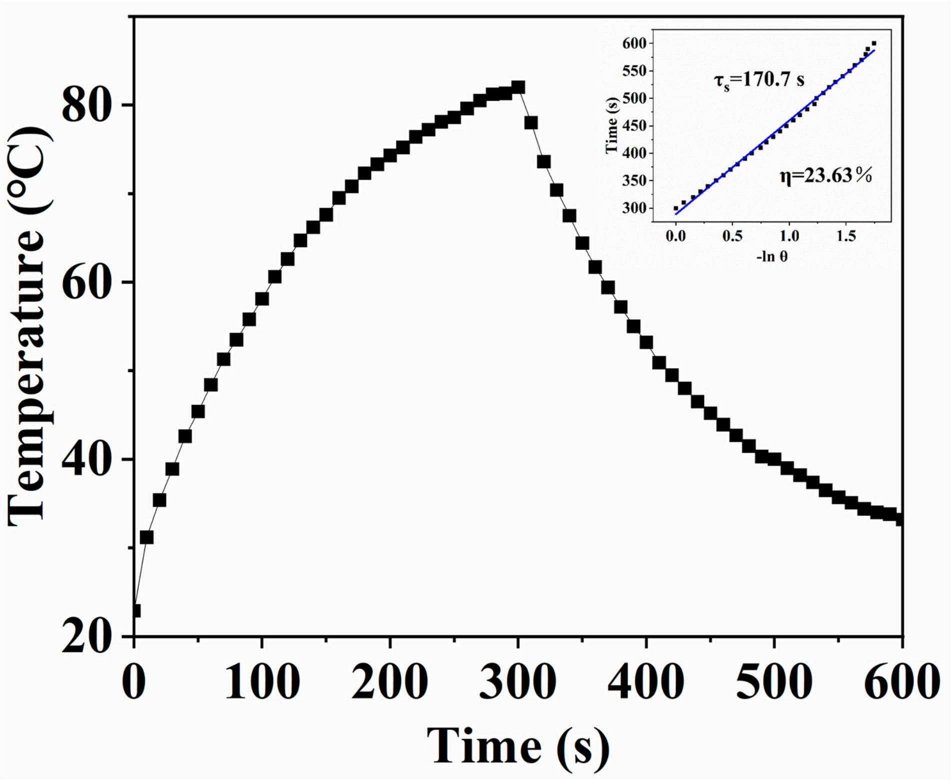

Figure S5. The photothermal conversion efficiency (η) of DSF@CuMSN-PDA (100 μg/mL) under 2.0 W/cm² laser irradiation with 808 nm.

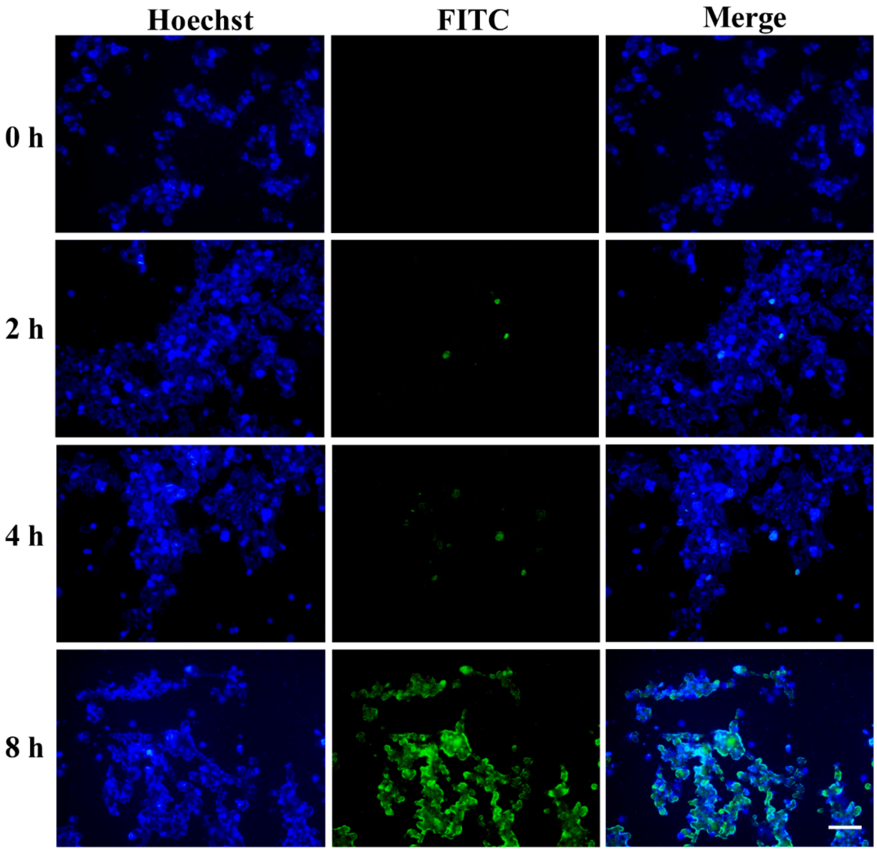

Figure S6. Fluorescence images of cellular uptake of FITC@CuMSN-PDA after 0 h, 2 h, 4 h, and 8 h incubation (scale bar: 100 μm).

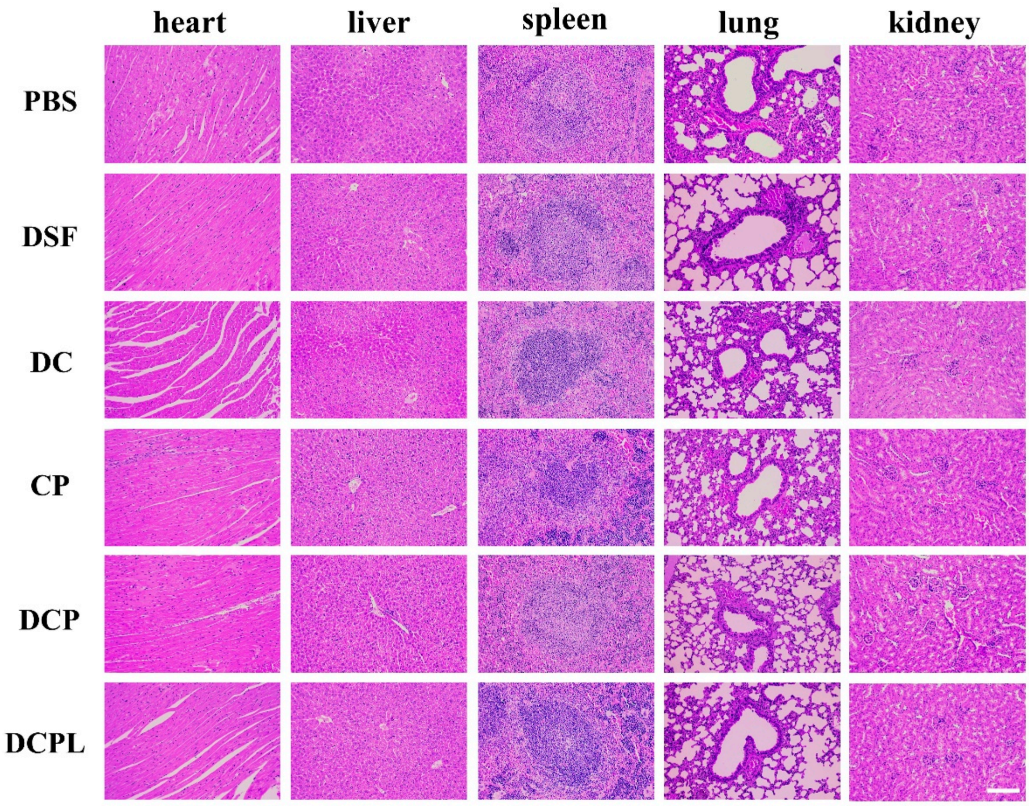

Figure S7. H&E staining of major organs after different treatments.
